# Integrated systems for biopolymers and bioenergy production from organic waste and by-products: a review of microbial processes

**DOI:** 10.1186/s13068-017-0802-4

**Published:** 2017-05-02

**Authors:** Giorgia Pagliano, Valeria Ventorino, Antonio Panico, Olimpia Pepe

**Affiliations:** 1 Division of Microbiology, Department of Agricultural Sciences, University of Naples Federico II, Via Università 100, Portici, 80055 Naples, Italy; 2 Telematic University Pegaso, Naples, Italy

**Keywords:** Biochemicals and bioenergy, Biopolymers, PHAs, Biogas, Biohydrogen

## Abstract

Recently, issues concerning the sustainable and harmless disposal of organic solid waste have generated interest in microbial biotechnologies aimed at converting waste materials into bioenergy and biomaterials, thus contributing to a reduction in economic dependence on fossil fuels. To valorize biomass, waste materials derived from agriculture, food processing factories, and municipal organic waste can be used to produce biopolymers, such as biohydrogen and biogas, through different microbial processes. In fact, different bacterial strains can synthesize biopolymers to convert waste materials into valuable intracellular (e.g., polyhydroxyalkanoates) and extracellular (e.g., exopolysaccharides) bioproducts, which are useful for biochemical production. In particular, large numbers of bacteria, including *Alcaligenes eutrophus*, *Alcaligenes latus*, *Azotobacter vinelandii*, *Azotobacter chroococcum*, *Azotobacter beijerincki*, methylotrophs, *Pseudomonas* spp., *Bacillus* spp., *Rhizobium* spp., *Nocardia* spp., and recombinant *Escherichia coli*, have been successfully used to produce polyhydroxyalkanoates on an industrial scale from different types of organic by-products. Therefore, the development of high-performance microbial strains and the use of by-products and waste as substrates could reasonably make the production costs of biodegradable polymers comparable to those required by petrochemical-derived plastics and promote their use. Many studies have reported use of the same organic substrates as alternative energy sources to produce biogas and biohydrogen through anaerobic digestion as well as dark and photofermentation processes under anaerobic conditions. Therefore, concurrently obtaining bioenergy and biopolymers at a reasonable cost through an integrated system is becoming feasible using by-products and waste as organic carbon sources. An overview of the suitable substrates and microbial strains used in low-cost polyhydroxyalkanoates for biohydrogen and biogas production is given. The possibility of creating a unique integrated system is discussed because it represents a new approach for simultaneously producing energy and biopolymers for the plastic industry using by-products and waste as organic carbon sources.

## Background

Over the past few decades, the need to reduce pollutant emissions produced by conventional systems of organic waste disposal has promoted the development of technologies that convert organic waste into bioenergy and biomaterials. In the near future, this new approach in waste management, in addition to being eco-friendly, can reasonably replace fossil fuels with biomass (organic waste or energy crops) as a source of both energy and materials (e.g., plastics) and therefore make two contributions toward reducing greenhouse gas (GHG) emissions into the atmosphere [1].

Petrochemical-derived materials can be replaced with biodegradable materials and biochemicals derived from renewable sources. In fact, organic waste materials are interesting renewable resources that can be converted into different value-added products, such as bioethanol or biochemicals obtained by sugar fermentation [2, 3]. Recent technological developments have explored the value of biochemical products as precursors to biopolymers, e.g., succinic acid [4, 5] and 2,3-butanediol [6] derived from lignocellulosic biomass. Some biopolymers can be produced by microorganisms from the accumulation of extracellular materials, such as exopolysaccharides (EPS) [7], and used in the food, chemical, cosmetic, and packaging industries as adhesives, absorbents, lubricants, and cosmetics. Furthermore, several biopolymers, such as polyhydroxyalkanoates (PHAs), polylactides, aliphatic polyesters, and polysaccharides [8], have already been successfully tested as bioplastics [9] because their physical and chemical properties perform just as well as conventional synthetic plastics. Among them, PHAs have gained much attention thanks to their complete biodegradability under various conditions within a period of 1 year [10]. Different bacteria (e.g., *Alcaligenes* spp., *Azotobacter* spp., methylotrophs, *Pseudomonas* spp., *Bacillus* spp., and recombinant *Escherichia coli*) have been used in PHA production from different low-cost substrates. In fact, to replace conventional petrochemical-derived plastics, useful substrates for PHA production include organic waste and by-products. In fact, to commercialize PHAs, substantial effort has been devoted to reducing the production cost through the development of bacterial strains and more efficient fermentation/recovery processes because the price of the substrate has the largest influence on the production cost of PHA [11].

To make PHA production more feasible for industrial application, future prospects are mainly focused on promoting less expensive substrates, improved microorganism cultivation strategies, and easier downstream processing methods, which are required for reducing production costs [12]. For this reason, different inexpensive substrates, such as molasses and sucrose, starch-based materials, cellulosic and hemicellulosic materials, sugars, whey, oils, fatty acids and glycerol, and organic matter from waste and wastewater [13], have been tested to produce biopolymers, and the results are promising.

Furthermore, it is important to highlight that the same substrates used to produce biopolymers represent a source of renewable energy (biomethane and biohydrogen) obtainable through an anaerobic digestion process. Therefore, such substrates can be simultaneously used to produce bioenergy and biopolymers, thus achieving a maximum valorization when they are used as organic waste.

The anaerobic digestion process is characterized by biochemical reactions in series carried out by different consortia of bacteria that convert organic compounds into methane, carbon dioxide, water, and ammonia. In the first step, complex and not negligibly sized biomolecules of organic materials are disintegrated and subsequently hydrolyzed into soluble, biodegradable organics by extracellular enzymes [14]. Then, acidogenic microorganisms metabolize products by hydrolysis into volatile fatty acids (VFAs) (acidogenic phase) [15]. Acidogenic products are first converted into acetic acid, hydrogen, and carbon dioxide (acetogenic phase) and, finally, into methane by methane-producing Archaea (methanogenic phase) [16]. The same substrates of methanogenic metabolism are the precursors that form PHAs [17]. Thus, this review gives insights into the current methodology for producing PHAs and biogas, with a focus on the use of organic waste and by-products as raw materials to keep production costs low. Moreover, this review examines the potential of several biological processes that can occur in the development of an innovative unique integrated system able to simultaneously produce bioenergy and biopolymers.

## Bio-based and biodegradable polymers: PHAs production and classification

Polyhydroxyalkanoates (PHAs) represent a group of bio-based and biodegradable polymers, considered similar to petroleum-based polymers [18].

Many bacteria, such as *Cupriavidus* (*C.*) *necator* [10, 19–27], different *Pseudomonas* (*P.*) species (*P. fluorescens, P. hydrogenovora, P. oleovorans, P. resinovorans, P. aeruginosa, P. mendocina*) [25, 28–32], strains belonging to *Azotobacter* (*A.*) species (*A. vinelandii, A. chroococcum*, *A. beijerinckii*) [33–37], *Bacillus* (*B.*) spp. [38–40], recombinant *Escherichia* (*E.*) *coli* [8, 12, 35, 41–44], and *Burkholderia* (*Bk.*) spp. [45, 46], synthesize PHAs as intracellular carbon and energy storage, accumulating these polyesters of hydroxyalkanoates as granules in the cytoplasm of cells [47]. Polyhydroxyalkanoic acids produced by bacteria are the building blocks of biodegradable thermoplastics and elastomers currently in use, or candidates to be used, in the medical and pharmaceutical industries as well as in agriculture [48]. The production of PHAs occurs mainly when cells are cultivated in the presence of a carbon source in excess, and their growth is limited by the lack of another nutrient, such as nitrogen, phosphorus, sulfur, or oxygen [49]. When the supply of the limiting nutrient is restored, PHAs are degraded by an intracellular depolymerase and subsequently metabolized as a carbon and energy source [50], and the number of bacteria rapidly increases.

Polyhydroxyalkanoates can be divided into two groups depending on the number of carbon atoms in the monomer units: short-chain-length (SCL) PHAs, which consist of 3–5 carbon atoms, and medium-chain-length MCL-PHAs, which consist of 6–14 carbon atoms [49]. The length of the side chain and functional group has great importance for the physical properties. The SCL-PHAs are crystalline, brittle, and stiff polymers, with a high melting point and a low glass transition temperature. In contrast, MCL-PHAs show low crystallinity and tensile strength and lower melting points.

**Fig. 1:**
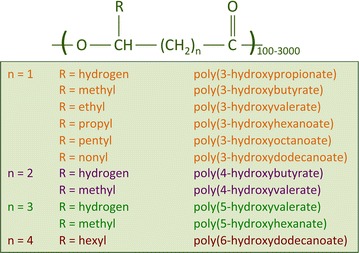
General structure of polyhydroxyalkanoates (PHAs). The most studied PHA type is the homopolymer P(3HB), for which *n* is equal to 1 and R is a methyl group [13]

Polyhydroxyalkanoates have the general formula shown in Fig. 1 [13], where “*n*” is equal to 1, and “R” is a methyl group. The most abundant PHA family member is poly(3-hydroxybutyrate) (P(3HB)). Using different substrates in a co-feeding system, copolymers of PHB (polyhydroxybutyrate) can be formed, such as polymers containing 3-hydroxyvalerate (3HV) or 4-hydroxybutyrate (4HB) monomers. 3HV can be incorporated into the PHB molecule, forming poly(3-hydroxybutyrate-co-3-hydroxyvalerate) [P(3HB-3HV)], resulting in a more brittle compound than P(3HB) [47].

Thus, to reduce the environmental footprint by producing and using petrochemical-derived products, they can be replaced partially and even completely by polyesters derived from biological processes (i.e., PHAs) that have the significant advantage of being completely biocompatible [47]. Biocompatibility is the property shown by certain materials that generates non-toxic compounds when they are disposed of after use as well having the same physical property of the artificial material derived from petrol that they would replace [13]. Unlike petroleum-derived plastics that take several decades to degrade, PHAs can be completely bio-degraded within a year by a variety of microorganisms, mainly bacteria and fungi [48]. In particular, several aerobic and anaerobic PHA-degrading bacteria, such as *Comamonas* sp. [51], *Pseudomonas lemoignei* [52] from soil, *Alcaligenes faecalis* [53] and *Pseudomonas fluorescens* from activated sludge [54] and *Pseudomonus stutzeri* from lake water [55], and fungi, such as *Aspergillus fumigatus* [54], have been isolated from various environments. These microorganisms excrete extracellular PHA depolymerases to degrade PHAs into water-soluble monomers and oligomers, using them as a carbon source (or methane under anaerobic conditions) [8].

Thus, life cycle assessment (LCA) conducted on the use of PHAs has been proven as the main advantage to avoid the accumulation of plastics in the environment [56]. Therefore, PHAs are better than petrochemical analogues, such as polyethylene and polypropylene [57–59], in terms of sustainability and environmental protection [60], but the realization and more widespread use of these environmentally friendly processes are related to the cost of the final product. The current PHA price also depends on monomer composition, and it is usually higher for copolymers; overall, it ranges from 2.2 to 5.0 € kg^−1^ [13, 61, 62], which is less than the typical range of 10–12 € kg^−1^ reported at the beginning of the past decade [61]. Notwithstanding the burden of costs and the environmental impacts of plastic trash, the current PHA prices are not deemed to be commercially competitive with respect to conventional petroleum-based polymers, which typically cost less than 1.0 € kg^−1^ [61, 63]. Although the price of PHAs is high, several companies are producing PHA products worldwide to meet the demand of the market, including in the UK, Japan, US, Germany, Brazil, Italy, and China [64, 65].

## Suitable substrates and bacterial strains for PHA production

The synthesis of PHAs occurs in many microorganisms under well-defined operating conditions and when they are supplemented with specific substrates, better known as PHA precursors. These compounds are incorporated into PHAs and used as the sole carbon source by microorganisms (or coupled with others as cosubstrates) if the cells are cultivated in the presence of an excess carbon source. Moreover, PHAs are also formed when growth is impaired or restricted by the lack of another nutrient, such as nitrogen, phosphorus, or oxygen [8]. Thus, different PHAs could be synthesized from the combination of different substrates and microorganisms under different growth conditions (aerobic or anaerobic, temperature, and pH).

Relevant substrates for the production of PHAs are as follows: carbon dioxide [66] or fossil resources, such as low-rank coal [67] renewable resources (e.g., starch [19, 20, 26, 38, 68], cellulose [69], sucrose [28, 33, 45]), waste materials (e.g., molasses [33, 39, 41], whey [12, 29, 42–44], glycerol [10]), and chemicals (e.g., propionic acid [70]). To avoid the use of fossil resources due to environmental issues and to limit PHA production costs, renewable resource and waste materials are reasonably considered suitable and promising substrates.

In the following paragraphs, an overview of different works categorized on the basis of the different substrates used is given. The results are presented in terms of the PHA content (%PHAs, %) and concentration ([PHAs], g l^−1^) calculated by the following Eqs. 1 and 2, respectively, where mPHA is the amount of PHAs (mg), mcells (mg) is the amount of freeze-dried biomass in samples, and CDW is the cell dry weight (g l^−1^):

1 $$\% {\text{PHAs}} = \frac{\text{mPHAs}}{\text{mcells}} \times 100$$

2 $$\left[ {\text{PHAs}} \right] = \frac{{\% {\text{PHAs}}}}{100} \times {\text{CDW}}.$$

### Starch-based material as a source of PHAs

Starch is a renewable carbon source available in large amounts. Prior to fermentation, starch is hydrolyzed to glucose by a two-step process, liquefaction and saccharification, because PHA-producing bacteria cannot synthesize amylase enzymes for starch degradation. Commercial hydrolyzing enzymes are often used, but they contribute to an increase in the cost of the glucose production process [35]. Kim [35] used soluble starch to produce P(3HB), obtained after 70 h of incubation with 25 g l^−1^ of PHB (content of 46% in cell dry weight), in fed-batch cultures of *A. chroococcum* strain 23 under oxygen-limiting conditions. Halami [38] reported the ability of the isolated strain *Bacillus cereus* CFR06 to accumulate PHAs in a starch medium composed of soluble starch, yeast extract, and salts. The genus *Bacillus* was identified as one of the first gram-positive bacteria suitable to produce PHAs and was cultivated under nitrogen limitation in Luria–Bertani (LB) broth for 24 h at 37 °C on a rotary shaker at 100–150 rpm min^−1^. The results obtained were less promising than those found by Kim [35] because, after 72 h of incubation, a P(3HB) concentration of 0.48 g l^−1^ with a content of 48% was achieved. Koutinas et al. [19] proved the potential of *C. necator* (synonym *Wautersia eutropha* and formerly classified as *Alcaligenes eutrophus,* formerly classified as *Ralstonia* (*R.*) *eutropha* [68]) in PHB production from a specific substrate derived from wheat. The authors conducted fed-batch tests using a 500-ml shake flask on a 250 rpm rotary shaker at 30 °C and a pH range of 6.5–6.8. The results showed a PHB concentration of 51.1 g l^−1^ using a culture medium with free amino nitrogen as substrate at a concentration of 1.2 g l^−1^. Under the same operating conditions (working volume, rpm, temperature, and pH range), Xu et al. [20] compared the batch and fed-batch modes using *C. necator* NCIMB 11599 grown on wheat-derived media. They demonstrated that more PHB was accumulated in cells operating in fed-batch mode. In fact, the use of fed-batch mode allowed for an increase in PHB concentration to 130.2 g l^−1^ (PHB cells content ~80%) compared with batch fermentation that showed a production of 41.5 g PHB l^−1^ (PHB cells content ~66%). Haas et al. [21] used saccharified waste potato starch as a carbon source for PHB production by *C. necator* NCIMB 11599, obtaining a PHB concentration of 94 g l^−1^, with a specific yield from starch of 0.22 PHB g starch g^−1^ under phosphate-limiting conditions. Poomipuk et al. [71] isolated and selected the strain *Cupriavidus* sp. KKU38, which was able to accumulate PHAs up to 65.27% (PHA concentration of 2.8 g l^−1^) from cassava starch hydrolysate as a sole carbon source in a 250-ml flask (Table 1).

**Table 1:** Overview of studies reporting PHA production from starch-based materials

Strain	Type of PHA	Operation mode	Time to PHA_max_ [h]	PHA concentration [g l^−1^]	PHA content [%]	References
*Azotobacter chroococcum* 23	P(3HB)	Fed-batch	70	25	46	[35]
*Bacillus cereus* CFR06	P(3HB)	Batch	72	0.48	48	[38]
*Cupriavidus necator* NCIMB 11599	P(3HB)	Fed-batch	168	51.1	70	[19]
*Cupriavidus necator* NCIMB 11599	P(3HB)	Batch	69	41.5	66	[20]
		Fed-batch	171	130.2	80	
*Cupriavidus necator* NCIMB 11599	P(3HB)	Batch	72	94	22	[21]
*Cupriavidus* sp. KKU38	PHA	Batch	96	2.8	65.3	[71]
Recombinant *E. coli* SKB99	P(3HB)	Batch	72	1.2	40	[72]

However, to overcome the high costs of the hydrolysis of starch into glucose by a two-step process (liquefaction and saccharification), making this feedstock less economically viable, Bhatia et al. [72] constructed the recombinant *E. coli* strain SKB99 harboring plasmids containing genes for starch hydrolysis (from *Paenibacillus* sp.) and PHB synthesis (from *R. eutropha*). This engineered strain utilized starch as the sole carbon source, with a maximum PHB production of 1.24 g l^−1^ (PHB content 40%) for 72 h with 2% (w/v) starch (Table 1). In addition, the accumulation of PHB started with the growth of the strain *E. coli* SKB99 and remained consistent until it attained the stationary phase, highlighting that PHB production in this engineered strain is not regulated by the stress response, unlike in *R. eutropha* and other microorganisms.

Therefore, starch-based materials are suitable substrates for PHA accumulation and, in particular, for P(3HB) accumulation. However, PHA accumulation strictly depends on the bacterial species and strains that exhibit different biotechnological performances depending on the carbon source and the culture conditions. The best results were obtained using *C. necator* NCIMB 11599 cultured on wheat and hydrolyzed waste potatoes under nutrient (nitrogen or phosphorus)-limiting conditions, operating in batch and fed-batch mode, respectively.

**Table 2:** Overview of studies reporting PHA production from molasses and sucrose

Strain	Type of PHA	Operation mode	Time to PHA_max_ [h]	PHA concentration [g l^−1^]	PHA content [%]	References
Recombinant *E. coli* (*HMS174/pTZ18u*-*PHB*) (*C. necator* genes)	P(3HB)	Fed-batch	31.5	31.6	80	[41]
*Pseudomonas fluorescens* A2a5	P(3HB)	Batch	96	22.4	70	[28]
*Cupriavidus necator*	P(3HB)	Fed-batch	45–50	80–100	65–70	[45]
*Bacillus cereus* M5	P(3HB)	Batch	72	0.13	73.8	[39]
*Azotobacter vinelandii* UWD	P(3HB)	Fed-batch	35	23	66	[33]
*Bacillus megaterium* BA-019	P(3HB)	Fed-batch	24	72.7	42	[73]

### Molasses and sucrose as sources for PHAs

Molasses is a common industrial by-product of sugar production, is much cheaper than glucose, and is extensively used as a carbon source for PHA production from biological processes. Liu et al. [41] demonstrated that recombinant *E. coli* (HMS174/pTZ18u-PHB) can efficiently utilize molasses as the sole carbon source to produce PHB. A fed-batch feeding strategy was developed to improve cell growth and PHB production. The final PHA concentration was 31.6 g l^−1^, and 80% of PHAs was accumulated (Table 2). Jiang et al. [28] isolated a strain of (PHB)-accumulating bacteria from the soil in Alaska (USA), identified as *P. fluorescens* A2a5. This microorganism is capable of accumulating a large amount of granules in its cells when grown in sugarcane liquor medium. Batch cultivation was carried out at 25 °C in a 5-l bioreactor inoculated with 1% inoculum (v/v) at pH 7.0. In this way, a maximum cell dry weight (CDW) of 32 g l^−1^ with a PHB concentration of 22.4 g l^−1^ was obtained, and the PHB content was approximately 70%. *C. necator* was aerobically grown in a well-balanced medium consisting of sugarcane and inorganic nutrients to reach a high cell density [45]. Then, cell growth was shifted to PHB synthesis by limiting nutrients other than the carbon source. The fed-batch fermentation process was carried out by continually feeding (45–50 h) a high concentration of sugar syrup to achieve a biomass of nearly 65–70% PHB, with a concentration ranging from 80 to 100 g l^−1^ (Table 2).

The effect of different molasses concentrations (1–5 g molasses/100 ml water) on PHB production by *B. cereus* M5 was investigated by Yilmaz and Beyatli [39]. They observed that PHB productivity by this strain decreased with increasing molasses concentration. In fact, the highest P(3HB) concentration produced by this strain was 0.1 g l^−1^ (polymer content of 73.8%) with 1% molasses concentration.

*Azotobacter vinelandii* UWD was investigated by Page et al. [33] using molasses as the sole carbon source. Fed-batch bioreactors were operated with 5% (w/v) molasses at pH 7.2 and inoculated with 4% (v/v) of the pre-grown strain. In the beet molasses medium, NH_4_ was depleted by 10–12 h to establish NH_4_-limiting conditions and fix nitrogen during the PHA production phase of growth. After 35 h, a P(3HB) concentration of 23 g l^−1^ and a polymer content of 66% were achieved.

Kulpreecha et al. [73] tested *B. megaterium* BA-019 on sugarcane molasses (20 g l^−1^) as a carbon source and urea or ammonium sulfate at 0.8 g l^−1^ as the investigated nitrogen sources. In the experiments, a cell dry mass concentration of 72.7 g l^−1^ in 24 h, with a PHB content of 42% (w/w), was achieved under nitrogen-limiting conditions operating in fed-batch mode (Table 2).

In addition, with sugarcane, *C. necator* showed the best PHA concentration among the bacterial strains (recombinant *E. coli*, *A. vinelandii* UWD, and *B. megaterium*) operating in fed-batch mode with molasses as a carbon source. In fact, *C. necator* is able to accumulate approximately 100 g l^−1^ synthesizing glucose (from starch) and sucrose (from sugarcane).

### Lignocellulosic material as a source for PHAs

To produce fuels and other valuable bioproducts, lignocellulosic biomass from dedicated crops and agricultural and forestry waste are promising renewable sources [74–77].

Lignocellulosic materials, consisting of lignin (complex polyphenolic structure), cellulosic (b-d-1,4-glucan), and hemicellulosic (d-arabinose, d-xylose, d-mannose, d-glucose, d-galactose, and sugar alcohols) fibers, constitute the most abundant renewable resources on our planet [13].

The composition of lignocellulosic biomass differs in terms of lignin (10–25%), cellulose (30–60%), and hemicellulose (25–35%) content [78].

Silva et al. [46] studied the potential of two bacterial strains, *Bk. cepacia* IPT 048 and *Bk. sacchari* IPT 101A, in producing P(3HB), comparing biosynthesis from xylose and glucose with bagasse hydrolysate. In high-cell-density cultures using a mixture of xylose and glucose under P limitation, both strains reached a maximum P(3HB) concentration of 60 g l^−1^ dry biomass, containing 60% biopolymer. Higher polymer content and yield were observed under P limitation than under N limitation for *Bk. sacchari* IPT 101A, whereas *Bk. cepacia* IPT 048 showed a similar performance in the presence of both growth-limiting nutrient conditions. Using bagasse hydrolysate as the carbon source, polymer contents reached 62 and 53% for *B. sacchari* IPT 101A and *B. cepacia* IPT 048, respectively, with a CDW of 4.4 g l^−1^ for both strains under N limitation (Table 3).

**Table 3:** Overview of studies reporting PHA production from lignocellulosic materials

Strain	Type of PHA	Operation mode	Time to PHA_max_ [h]	PHA concentration [g l^−1^]	PHA content [%]	References
*Burkholderia sacchari* IPT 101	P(3HB)	Batch	25	2.73	62	[46]
*Burkholderia cepacia* IPT 048	P(3HB)	Batch	25	2.33	53	[46]
*Cupriavidus necator*	PHA	Batch	48	3.9	65	[22]
Recombinant *E. coli* (*C. necator* genes)	P(3HB)	Batch	60	1.7	35.8	[69]
			60	4.4	73.9	
*R. eutropha* ATCC 17699 (*C. necator*)	P(3HB)	Batch	48	11.4	75.5	[79]

Yu and Stahl [22] also studied the performance of *C. necator* with the same substrate. In their experiment, the cultures were shaken in flasks at 200 rpm and 30 °C for 48 h with pH adjusted to approximately 7.5. They demonstrated that P(3HB) was the predominant biopolyester formed from the hydrolysis of sugarcane bagasse, with a concentration of 3.9 g l^−1^, corresponding to a P(3HB) accumulation of 65% of the CDW, achieved with a high carbon-to-nitrogen ratio (*C*/*N* = 20 or above). Since a minimum nitrogen level should be maintained during cultivation, this high *C*/*N* ratio implies a high concentration of residual organic carbon or a high amount of hydrolysates. A moderate *C*/*N* ratio (7–10) may be used to yield a low concentration (less than 1 g l^−1^) of residual carbons and a moderate level of PHA content in the cells (45–50% w/w).

Lee et al. [69] investigated P(3HB) production from xylose and hydrolyzed cellulose by growing recombinant *E. coli* strains with *C. necator* PHA biosynthesis genes, testing the effects of supplementing a complex nitrogen source on cell growth and PHB production. The cells were cultivated for 60 h in a 250-ml flask containing 50 ml of medium in a shaking incubator at 250 rpm. When the strain TG1 (pSYL107) was grown on 20 g l^−1^ xylose, it was capable of accumulating 1.7 g l^−1^ of P(3HB) with 35.8% of polymer content. A higher P(3HB) concentration, equal to 4.4 g l^−1^, and a polymer content of 73.9% were reached when the previous culture medium was supplemented with 10 g l^−1^ of soybean hydrolysate. To evaluate the effects of the nitrogen source, tryptone and peptone were also tested, achieving 47.7 and 10.3% of PHB content, respectively.

The ability of *R. eutropha* ATCC 17699 (*C. necator*) to produce PHB in the presence of different waste biomass hydrolysates (rice paddy straw, soybean husk, sunflower husk, and wood straw) was evaluated by Saratale and Oh [79]. The most suitable substrate for PHB accumulation by this strain was the rice paddy straw hydrolysate, which was selected by the authors for optimization of the process, obtaining the maximal PHA accumulation (75.45%) and PHB production (11.42 g l^−1^) within 48 h of fermentation.

Moreover, lignin and its derivatives are also used for PHA production. Tomizawa et al. [80] tested PHA-accumulating strains on mineral salt media containing each of the 18 lignin derivatives and hydroxybenzoic acids, including intermediates derived from the metabolism of lignin derivatives in bacteria. Most of the strains grew poorly in media containing lignin derivatives, such as p-coumaric acid, caffeic acid, ferulic acid, and sinapinic acid.

**Table 4:** Overview of studies reporting PHA production from whey-based culture media

Strain	Type of PHA	Operation mode	Time to PHA_max_ [h]	PHA concentration [g l^−1^]	PHA content [%]	References
Recombinant *E. coli* (*C. necator* genes)	P(3HB)	Batch	49	5.2	81.3	[42]
Recombinant *E. coli* (*C. necator* genes) GCSC 6576	P(3HB)	Fed-batch with oxygen limitation	52	25	80	[35]
		Fed-batch without oxygen limitation	35	32	57	
*Pseudomonas hydrogenovora* DSM 1749	P(3HB)	Fed-batch	41	1.27	12	[29]
	P(3HB-co-3HV)		31	1.44	12	
Recombinant *E. coli* K24 K (*Azotobacter* spp. genes)	P(3HB)	Fed-batch	24	51.1	72.9	[43]
Recombinant *E. coli* CGSC 4401	P(3HB)	Fed-batch	36.5	96.2	80.5	[12]
Recombinant *E. coli* CGSC 4401 (*A. latus* genes)	P(3HB)	Fed-batch 30-l bioreactor	26	35.5	70	[44]
		Fed-batch 300-l bioreactor	20	20	67	

On the contrary, *R. eutropha* PHB-4 accumulated P(3HB) from 3-hydroxybenzoic acid and 4-hydroxybenzoic acid as the sole carbon sources, with a PHA content of 65 and 63 wt% and a dry cell weight of 1.6 and 0.69 g l^−1^, respectively.

Although *C. necator* species seems to be the best bacterial candidate for PHB production using lignocellulosic hydrolysate, the accumulation is lower than that obtained with sucrose- and starch-based materials as carbon sources. The lowest PHA accumulation could be due to the presence of specific toxic compounds (e.g., furfural, HMF, *p*-hydroxybenzoic aldehyde, and vaniline) that are usually released during the pretreatment of lignocellulosic biomass, which are known to have an inhibitory effect on microbial growth and metabolism.

**Table 5:** Overview of studies reporting PHA production from oil, fatty acid, and glycerol culture media

Strain	Type of PHA	Operation mode	Time to PHA_max_ [h]	PHA concentration [g l^−1^]	PHA content [%]	References
*C. necator* DSM 545	P(3HB)	Fed-batch	33.5	51.2	62	[10]
*C. necator* H16 (ATCC 17699)	P(3HB)	Batch	72	2.9–3.4	79–82	[23]
*C. necator* H16 (pJRDEE32d13)	P(3HB)	Fed-batch	96	85–95	72–76	[24]
*C. necator* H16	P(3HB)	Batch	96	1.24	19.7	[25]
*Pseudomonas resinovorans*	PHA	Batch	48	0.14	15.2	[30]

### Whey-based culture media as a source for producing PHAs

Whey is the major by-product of cheese factories, representing 80–90% of the volume of transformed milk [42]. It contains approximately 4.5% (w/v) lactose, 0.8% (w/v) protein, 1% (w/v) salts, and 0.1–0.8% (w/v) lactic acid, and its high biological oxygen demand (40 g l^−1^) makes it difficult to dispose. The discharge of large amounts of cheese whey into the environment can damage the chemical and physical structure of soil and pollute groundwater and can also affect the air [81]. This by-product represents an attractive low-cost substrate for producing PHAs.

As seen in the previous sections, *C. necator* is one of the best-known bacteria among PHA-producing microorganisms, but it is unable to hydrolyze lactose or metabolize galactose [82]. In fact, *C. necator* was able to use lactose only after the expression of genes encoding β-galactosidase and galactokinase, although at a very slow rate [83]. Therefore, recombinant *E. coli* containing the *C. necator* PHA biosynthesis genes for the production of PHB from glucose is considered a good candidate for PHB production from whey [42]. Lee et al. [42] cultivated recombinant *E. coli* strains in a defined medium supplemented with varying concentrations of whey solution and obtained 5.2 g l^−1^ of PHB, corresponding to 81.3% (w/w) of PHB, with a concentration of 30 g l^−1^ of whey solution (Table 4). Kim [35] also studied recombinant *E. coli* strains as PHB-accumulating microorganisms under O_2_ limitation compared with conditions without O_2_ limitation. The highest PHB accumulation (80%) was observed under O_2_-limiting conditions, with a PHB concentration of 25 g l^−1^. Instead, without O_2_ limitation, 57% of PHB was achieved with a concentration of 32 g l^−1^. A recombinant *E. coli* strain containing the PHA biosynthetic genes from *Azotobacter* spp., specially designed for the production of PHB from milk whey, was studied by Nikel et al. [43]. Fed-batch cultures were carried out at 37 °C in a 5.6-l fermentor with a starting volume of 2.0 l and a controlled pH of 7.20. The feeding solution used for fed-batch cultures was a concentrated and deproteinated whey solution containing 25% (w/v) lactose. They reported that after 24 h, the cells accumulated PHB up to 72.9% of their cell dry weight, reaching a PHA concentration of 51.1 g l^−1^. Physical analysis of PHB collected from the recombinants showed that its molecular weight was similar to PHB produced by an *Azotobacter* spp. strain.

A new fermentation strategy using a cell recycle membrane system was developed by Ahn et al. [12] for the efficient production of P(3HB) from whey by a recombinant *E. coli* strain harboring the *Alcaligenes latus* PHA biosynthesis genes.

Cell fed-batch cultures of recombinant *E. coli* CGSC 4401 (pJC4) were carried out to overcome the volumetric limitation of a fermentor (2.7 l) fed with a solution with low lactose solubility to increase PHB productivity. A whey solution containing 210 g lactose l^−1^ was used as a feeding solution. The final cell concentration, PHB concentration, and PHB content obtained in 39 h were 150, 100 g l^−1^, and 67%, respectively. In another experiment, a whey solution containing 280 g lactose l^−1^ was used as a feeding solution. After 36.5 h, a PHB concentration and a PHB content of 96.2 g l^−1^ and 80.5%, respectively, were obtained using a whey solution concentrated to contain 280 g lactose l^−1^ as a feeding medium. No inhibitory effects of the by-products or nutrients on cell growth and PHB production were found during fermentation by the authors.

The production of P(3HB) from whey by fed-batch cultures of recombinant *E. coli* harboring a plasmid containing the *Alcaligenes latus* PHA biosynthesis genes was examined by Park et al. [44]. Fed-batch cultures of recombinant *E. coli* SGSC 4401 (pJC4) were carried out at 30 °C in 30 l (working volume of 10 l) and 300 l (working volume of 150 l) fermenters supplying only air. The culture pH was controlled at 6.9. With lactose below 2 g l^−1^, the cells grew to 12 g l^−1^ with 9% (w/w) P(3HB) content in a 30 l fermenter. The accumulation of P(3HB) could be triggered by increasing lactose to 20 g l^−1^. Using this strategy, 35.5 g l^−1^ was obtained with a 70% (w/w) P(3HB) content after 26 h. The same fermentation strategy was used in a 300 l fermenter, and a 20 g l^−1^ with 67% (w/w) P(3HB) content was obtained in 20 h by Park et al. [44]. Koller et al. [29] compared the production of PHB under nitrogen-limiting conditions obtained with *P. hydrogenovora* using the following two substrates: hydrolyzed whey permeate and glucose/galactose medium. Shake flasks (1 l) containing 250 ml of hydrolyzed whey permeate or synthetic medium supplemented with glucose and galactose (each 2.5 g l^−1^) were both inoculated with 5% (v/v) *P. hydrogenovora*. The flasks were shaken at 30 °C for 48 h. Furthermore, the study investigated the influence of the 3HV precursor sodium valerate on the bacterial growth of *P. hydrogenovora.* Thanks to its advanced properties compared with those of highly crystalline pure PHB [29], the ability of the strain to biosynthesize P(3HB-co-3HV) in media supplemented with hydrolyzed whey permeate and sodium valerate was evaluated. In these two different experiments, PHA content was confirmed at 12% for both types of PHAs, but the PHA concentration was higher when sodium valerate was added to P(3HB-co-3HV) production (Table 4).

A recombinant strain of *E. coli* was generally used to obtain the PHA concentration (more than 90 g l^−1^) from whey-based culture media because *C. necator* is unable to hydrolyze lactose. In fact, several studies tested different lactose concentrations to correlate this parameter to PHA accumulation. Fed-batch experiments supplemented with a high amount of lactose (hydrolyzed from chees whey) were performed to obtain a higher PHA concentration. Otherwise, when increasing the lactose concentration to 280 g l^−1^, a relevant increase in PHA concentration was not observed.

In addition, it is interesting to note that with whey-based culture media, the oxygen-limiting conditions enhance PHB biosynthesis from recombinant *E. coli* but decrease PHA concentration in the cells.

### Fatty acid and glycerol culture media as source for PHAs

Pure glycerol is an important industrial feedstock, with applications in the food, drug, cosmetic, and tobacco industries, while crude glycerol is the main by-product of biodiesel production, with low value due to the presence of impurities (such as methanol, salts, and fatty acids). Thus, crude glycerol represents a waste product with an associated disposal cost [10]. For this reason, it can be used as an attractive substrate for PHA production.

*Cupriavidus necator* DSM 545 was used by Cavalheiro et al. [10] to accumulate P(3HB) from waste glycerol and from commercial glycerol as a control substrate. For *C. necator* cultivated on basal medium supplemented with pure glycerol and nitrogen depletion, a maximum of 51.2 g l^−1^ of P(3HB) at 33.5 h was reached, with a PHB content of 62% (Table 5). On the contrary, using waste glycerol as a carbon source, productivity was lower because only 38.5 g l^−1^ was achieved with a PHB content of 50% in 34.5 h.

Production of PHAs from various plant oils or oleic acid by *C. necator* H16 was studied by Fukui and Doi [23]. The strain was tested on olive oil, corn oil, and palm oil and in all these plant oils. The strain was cultivated in a 100-ml nitrogen-limited mineral salt medium containing 1% plant oil at 30 °C for 72 h. The wild-type strain produced P(3HB) at a high polymer content (79–82%) but at low concentrations (2.9–3.4 g l^−1^).

Kahar et al. [24] produced a copolymer of 3HB with 5 mol% (R)-3-hydroxyhexanoate, P(3HB-co-3HHx), from soybean oil as a sole carbon source with a recombinant strain of *C. necator*. The medium for PHA production in the fermentor was a mineral salt medium, and the initial concentration of NH_4_Cl was set at 4 g l^−1^. Additional NH_4_Cl was intermittently fed into the culture broth to avoid nitrogen source depletion. Soybean oil was added to the fermentor for an initial concentration of 20 g l^−1^. A high content of P(3HB) (85–95 g l^−1^) and a high PHA content of 71–74% (w/w) were achieved during 96 h.

Füchtenbusch et al. [25] studied *R. eutropha* and *P. oleovorans* cultivated in a mineral salt medium with the oil from rhamnose production as the sole carbon source under aerobic conditions at 30 °C in nutrient broth or in mineral salt medium.

The concentration of ammonium was limited to 0.05% (m/v) to promote the accumulation of PHAs. The cultivation of *P. oleovorans* and *R. eutropha* was performed in 300 ml at 28 °C. *C. necator* accumulated only P(3HB) at 6.3 g l^−1^, with a polyester content of 19.7% during the first 96 h (Table 5). The same authors tested *P. oleovorans* under the same operating conditions using the same carbon source. After 96 h, this strain accumulated 5 g l^−1^, with a P(3HB-co-3HHx) content of 17.3%.

Different *Pseudomonas* species (*P. oleovorans*, *P. resinovorans*, *P. putida*, and *P. citronellolis*) were tested by Cromwick et al. [30] in 2-l shake flasks. The bacteria were evaluated for their ability to grow and produce PHAs using tallow-free fatty acids and tallow triglyceride as carbon substrates; however, only *P. resinovorans* was able to grow and produce PHAs. The PHA concentration in this case was 0.12–0.15 g l^−1^, with a 15.2% polymer content, using unhydrolyzed tallow as the substrate.

The different fatty acids and glycerol waste materials used as substrates for PHA accumulation highlighted that *C. necator* was the best candidate operating under nitrogen source depletion, although PHA accumulation depended on the strain and operating mode. In fact, performing the experiments in fed-batch mode, more PHB was accumulated in the cells than operating in batch mode.

### Solid agro-industrial by-products and waste as a source of PHA production

Law et al. [40] showed that recombinant *B. subtilis* could utilize malt waste in the medium as a carbon source better than glucose and thus could substantially lower the cost of PHA production. In the paper by Law and co-authors, the *pha* genes (involved into PHA accumulation) from *B. megaterium* were cloned into *B. subtilis*. The recombinant strain was cultivated by acid hydrolyzed malt waste, and a 1% inoculum was used in a fermentation flask incubated at 37 °C at 280 rpm for 16 h. Their results showed PHA accumulation in a malt waste medium of 2.53% with a PHB concentration of 0.06 g l^−1^ in 12 h (Table 6).

*Aztoobacter vinelandii* UWD strains were tested by Cho et al. [34] with most poly-3-hydroxybutyrate-co-valerate (PHBV) production from swine waste liquor. Strain UWD was cultured in a shake flask with 4% inoculum at 200 rpm, incubated at 30 °C for 18–52 h.

Using undiluted swine waste liquor medium without glucose supplementation, cell growth was limited to 1.2 g l^−1^ with 37% in 48 h. Cell growth and PHBV production increased when swine waste liquor was diluted twofold and supplemented with 30 g glucose l^−1^ (5.48 g l^−1^ and PHBV content 58%).

Industrial fruit and vegetable waste were successfully used as sole carbon sources by Ganzeveld et al. [84] to produce PHBV by *R. eutrophus* under oxygen-limiting conditions. The fermentor was a 1-l standard fermentor with a working volume of 750 ml. The temperature was controlled at 30 °C. The stirrer speed was adjusted manually to maintain the dissolved oxygen pressure above 30% of the saturation concentration. A concentration of 1.1 g PHBV l^−1^, or 40% (w/w) of the cell dry weight, was obtained.

Starchy wastewater was used by Yu [26]. The waste was first digested in a thermophilic upflow anaerobic sludge blanket (UASB) reactor to form acetic, propionic, and butyric acids. PHA formation from individual acids was further investigated under nitrogen-limiting conditions by active biomass of *R. eutropha*. PHA formation from acid effluent in 48 h was 1.2 g l^−1^, with a PHA content of 34.1% (Table 6).

Another suitable substrate for PHA production is food scraps, a complex form of organic solid waste that is unusable by PHA-producing microbes, such as *R. eutropha*. Hydrolysis and acidogenesis are the main processes used to convert biodegradable solids into short-chain volatile fatty acids, such as acetic, propionic, and butyric acids, which are utilized by PHA-producing bacteria. This approach was used by Du et al. [85] by coupling organic acid production with anaerobic acetogenesis to produce PHAs. The PHA-synthesis reactor (2-l air-bubbling bioreactor) was maintained at 30 °C via a water jacket and pH 7.5. The dissolved oxygen concentration was maintained at 20% of air saturation or above. The PHA content and concentration reached their maximal values of 72.6% and 16.5 g l^−1^, respectively, in 73 h.

**Table 6:** Overview of studies reporting PHA production from solid agro-industrial by-products

Strain	Type of PHA	Operation mode	Time to PHA_max_ [h]	PHA concentration [g l^−1^]	PHA content [%]	References
Recombinant *Bacillus subtilis* 1A304 (105 MU331)	P(3HB)	Batch	12	0.06	2.5	[40]
*Azotobacter vinelandii* UWD (ATCC 53799)	P(3HB-co-3HV)	Batch	48	0.43	37	[34]
			18	5.48	58.3	
*C. necator* (*R. eutropha*)	P(3HB-co-3HV)	Batch	45	1.13	40.8	[84]
		Batch	48	1.2	34.1	[26]
		Fed-batch	73	16.5	72.6	[85]
Activated sludge	P(3HB)	Batch	96	2.7	67	[87]

Other studies were conducted on the use of excess activated sludge from a wastewater treatment plant fed with industrial waste streams as a substrate for PHB accumulation [86]. Wastewater from food processing (producing mainly potato chips, wafers, and sweets) and starch-rich grain-based alcohol industries (rice grain-based and jowar grain-based distillery spent wash) was used as a substrate for PHB production by Khardenavis et al. [87]. In their work, different types of wastewater were tested in 250-ml conical flasks and incubated on a rotary shaker at 150 rpm at 30 °C: wastewater derived directly from industry, filtered wastewater, and deproteinized wastewater, each in the absence and presence of an external nitrogen source; the highest biomass concentration of 6.6 g l^−1^ (dry weight) was produced in 96 h in a raw rice grain-based distillery spent wash with the addition of di-ammonium hydrogen phosphate, accumulating 2.7 g l^−1^ PHB with a content of 67%; a deproteinized jowar grain-based distillery spent wash and filtered food processing wastewater yielded lower PHB and biomass accumulation.

The studies carried out using solid agro-industrial by-products and waste demonstrated that the accumulation of PHAs was lower than that obtained with the other complex starting matrices, which was also observed when lignocellulosic hydrolysates were used as carbon sources. In addition, with this organic biomass, the highest accumulation was achieved using *C. necator* species, although the operating mode strongly influenced the process. Interestingly, activated sludge from a wastewater treatment plant was used as mixed cultures for PHA production from industrial waste streams. In particular, the PHA concentration was similar to that observed with pure cultures, overcoming the high costs derived from the production of pure cultures and the disposal of waste activated sludge.

## Integrated systems to simultaneously produce intracellular (PHAs) and extracellular by-products (biosurfactants)

Bacterial strains actively involved in PHA accumulation can be used at industrial scale to reduce the production costs of biopolymers due to their ability to convert waste materials into valuable intracellular and extracellular bi-products (e.g., PHAs and exopolysaccharides (EPS), respectively) that are useful for biochemical production. PHAs represent intracellular carbon and energy storage, while EPS and biosurfactants are produced as extracellular substances to protect the cells from desiccation and predation or are a carbon source. These substances are of industrial interest for washing powders and fabric softener production [88]. They are used also in the food, chemical, cosmetic, and packaging industries as adhesives, absorbents, lubricants, and cosmetics [89–91].

Biosurfactants are amphipathic molecules with polar and non-polar heads produced by different bacterial genera (e.g., *Acinetobacter*, *Arthrobacter*, *Bacillus*, *Pseudomonas*, *Rhodococcus*, and *Enterobacter*) [92]. Biosurfactants present as a wide variety of structures because their synthesis is influenced by the carbon source [93]. In fact, they can be produced on different substrates, such as sugars, lipids, alkanes, and waste materials [92]. The main property of biosurfactants is the ability to reduce surface and interfacial tension, forming microemulsions [94]. Among biosurfactants, rhamnolipids are the most studied thanks to the simultaneous production of PHAs and rhamnolipids by *P. aeruginosa* IFO3924 [31]. In their work, batch cultivation was conducted at 30 °C in 3-l fermentors equipped with an agitator using 7 g l^−1^ of decanoate as a carbon source. In this experiment, basal salt medium was used to increase the concentration of the nitrogen source. After a 3-day cultivation, considerable PHA content (23% of CDW corresponding to a concentration of 2.2 g l^−1^) and rhamnolipid amounts (298 mg l^−1^) were produced.

Another type of extracellular polymeric substance is EPS, a mixture of high molecular polymers, which supplies carbon units when substrate is limited. Wang and Yu [27] studied the simultaneous biosynthesis of EPS (an extracellular product) and PHB (an intracellular product) by *R. eutropha*. They observed that EPS production was closely coupled with cell growth, while PHB was synthesized only under nitrogen-limiting conditions and cell growth-limiting conditions. In fact, the experiments were conducted at different concentrations of glucose and NH_4_-N to evaluate their influence on EPS and PHB production. Furthermore, the previous authors observed that the PHB content in dry cells decreased with increasing nitrogen concentration, while the EPS concentration increased. While keeping the nitrogen concentration constant, further experiments were conducted at varying glucose concentrations, and the results showed that an increase in glucose concentration promoted biomass growth and PHB production. The relevant production (shown in Table 7) of both polymers was observed when glucose and nitrogen were supplied at concentrations of 40 and 3 g l^−1^, respectively.

**Table 7:** Overview of studies reporting PHA production coupled to metabolites used in industry

Strain	Type of PHA	Operation mode	Time to PHA_max_ [h]	PHA concentration [g l^−1^]	PHA content [%]	Produced metabolites [g l^−1^]	References
*Pseudomonas aeruginosa* IFO3924	PHA	Batch	72	0.5	23	Rhamnolipids 0.3	[31]
*Ralstonia eutropha* ATCC 17699	PHB	Batch	60	12.7	62	EPS 0.18	[27]
*Azotobacter beijerinckii* WDN-01	PHB	Batch	40	2.73	54.6	EPS 1.2	[37]
*Azotobacter chroococcum* 6B	PHB	Batch	48	0.74	28	EPS 2.1	[36]
*Pseudomonas mendocina* NK-01	PHA_MCL_	Batch	48	0.316	25.3	Alginate oligosaccharides 0.57	[32]

Among EPS, alginates are of great commercial interest for their use in a wide range of applications in the food industry, such as in frozen custards, restructured foods, cream and cake mixtures, and beer production. They are composed of variable amounts of β-d-mannuronic acid and C5-epimer α-l-guluronic acid linked via β-1,4-glycosidic bonds. When extracting alginates from harvested material, the uronic acids are converted into the salt forms mannuronate and guluronate through a neutralization step. The proportion, distribution, and length of these blocks determine the chemical and physical properties of the alginate molecules. Commercial alginates are currently extracted from marine algae, such as *Laminaria* and *Macrocystis*, but can also be obtained from bacterial species, such as *A. vinelandii*, *P. aeruginosa*, and *P. mendocina* [31]. The co-production of alginates and PHAs by *P. mendocina* using glucose as a carbon source was studied by Guo et al. [32]. The simultaneous production of MCL-PHA and alginate oligosaccharide (AO) cultivation was performed in 200 l fermenters with 120 l mineral salt medium containing 20 g l^−1^ glucose at 30 °C and 200 rpm of impeller speed for 48 h. The authors reported that 0.316 g l^−1^ PHA_MCL_ and 0.57 g l^−1^ AO were obtained at the end of the fermentation process. The MCL-PHA production reached a maximum of 0.360 g l^−1^ at 36 h when the carbon source was almost exhausted. At 48 h, the utilization of intracellular stored MCL-PHA took place, corresponding to a decrease in PHA content to 0.316 g l^−1^.

Moreover, the production of PHB and EPS by *A. beijerinckii* was investigated by Pal et al. [37] under nitrogen-free conditions with an excess of carbon. This strain was maintained by growth on nitrogen-free glucose medium at 30 °C for 48 h and was then stored at 4 °C. Nitrogen-free liquid medium was inoculated with 4% (v/v) inoculum, and the flasks were incubated at 30 °C on a rotary shaker. The highest production of PHB (2.73 g l^−1^) was reached when glucose was supplemented at 3% (w/v), observing an EPS amount of 1.2 g l^−1^.

Quagliano and Miyazaki [36] studied the simultaneous production of PHB and EPS by *A. chroococcum,* evaluating the influence of ammonium addition with glucose, fructose, and sucrose. The organism was grown aerobically in 250- and 500-ml flasks at a one-third volume of the culture medium with the carbon sources alone or supplemented with 0.1 g l^−1^ of (NH_4_)_2_SO_4_. The flasks were incubated in a rotatory shaker at 220 rpm at 30 °C for 72 h.

The highest PHB content was obtained with sucrose (1.1 g l^−1^), but EPS production was almost unobservable. Instead, the experiments conducted with glucose showed a maximum EPS concentration (2.1 g l^−1^), with PHB production of 0.74 g l^−1^.

Thus, some microorganisms, such as *P. aeruginosa, R. eutropha, A. beijerinckii, A. chroococcum*, and *P. mendocina*, are able to concurrently produce PHAs and biosurfactants using the same type of organic substrate. However, the bacterial technological performance during the coupled process of PHA and biosurfactant production leads to a lower accumulation of PHAs. In particular, the optimal operating conditions for PHA and biosurfactant production are different. In fact, Wang and Yu [27] observed that without nitrogen-limiting conditions, the PHB content in dry cells decreased, whereas the EPS concentration increased, demonstrating that nutrient-limiting conditions promote only PHA accumulation.

## Bioenergy production from industrial and agricultural waste

### Anaerobic digestion and biogas production

Anaerobic digestion is a consolidated biological treatment, mainly used for reducing organic content in the sludge produced from municipal wastewater treatment plants, thus achieving its stabilization [95]. In the past few decades, the need to drastically reduce the use of landfills for the disposal of organic waste and producing energy from renewable resources has promoted the use of anaerobic digestion for treating a wide range of organic solids, e.g., organic waste and energy crops [96, 97]. To calculate bioenergy production potential based on anaerobic digestion for biomethane, official data for food waste generation and management were collected by Dung et al. [98] from 21 countries, evaluating a methane potential equal to 379.769 kWh year^−1^.

Treatment systems based on the anaerobic digestion process are flexible because they can have different configurations according to the number of stages (one or two stages); can operate at different temperatures, mostly at 35 °C (mesophilic) or 55 °C (thermophilic); can be fed in batch, semi-batch, or continuous; can take place in completely stirred or plug flow reactors; and can work with a content of solids lower than 10% in mass (wet system) or higher than 20% (dry system), preceded by several innovative pretreatments to increase waste solubilization [99].

**Fig. 2:**
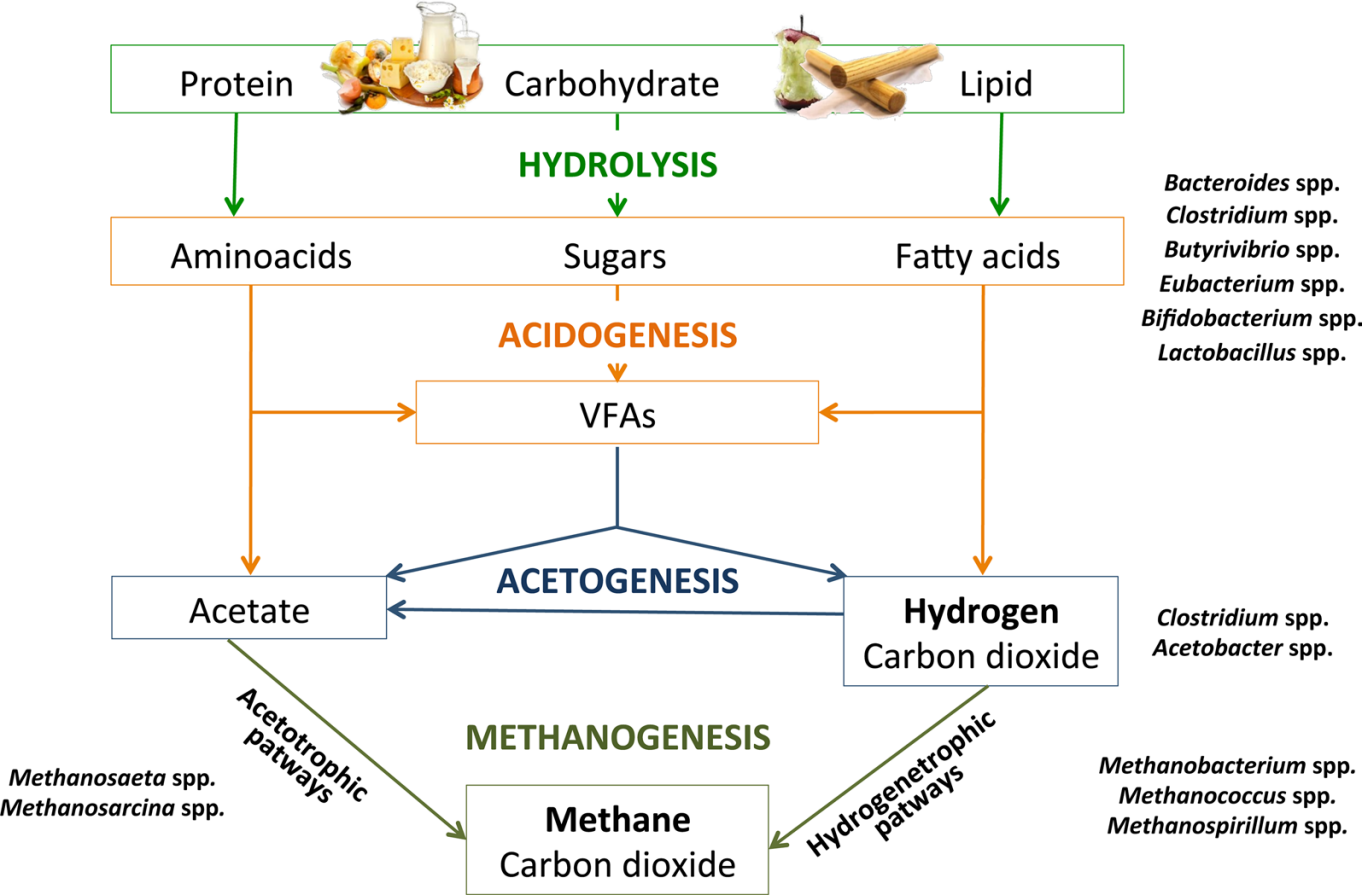
Phases of biological production of methane with the occurrence of VFAs, acetate, hydrogen, and carbon dioxide. Anaerobic bacteria involved are positioned according to their probable role in the process

Treating organic waste through anaerobic digestion results in economic and environmental advantages [97–100]; after treatment, the waste material is reduced in quantity, and it is more stable and less harmful for the environment because it is a source of a renewable energy, e.g., biogas, that does not alter the balance of CO_2_ in the atmosphere and therefore does not contribute to global warming [101]. Additionally, biogas refined to biomethane is also used to feed gas networks [102] as a surrogate to natural gas, and finally, the by-product of anaerobic digestion, named digestate, can be reused in agriculture as fertilizer [103, 104] thanks to its relevant content of nutrients. The performance and results of anaerobic digestion are strictly dependent on the environmental conditions [105–108], such as temperature, pH, nutrients content, presence of inhibitors [107], substrate composition and particle size, micronutrient availability, and the microbial strains used as the inoculum. Anaerobic digestion is driven by a complex microbiome containing both bacteria and Archaea. Each trophic group in the microbiome contains different microorganisms involved in different metabolic tasks [109]. A strong syntrophic relationship exists between different consortia of microorganisms, since biochemical reactions in series are carried out (Fig. 2). Bacteria are crucial in the hydrolyzation and acidogenic step of the anaerobic digestion process.

Novaes [110] reported that the anaerobic species belonging to the families *Streptococcaceae* and *Enterobacteriaceae* as well as the genera *Bacteroides*, *Clostridium*, *Butyrivibrio*, *Eubacterium*, *Bifidobacterium*, and *Lactobacillus* are most commonly involved in the anaerobic digestion process. Furthermore, during the process, bacteria, such as *Clostridia*, fermented the hydrolyzed products of proteins to VFAs, CO_2_, and hydrogen (H_2_).

In addition, Archaea are important in the methanogenic phase of anaerobic digestion. Methanogenic Archaea are strictly anaerobic and are able to transform fermentation products into CH_4_ [111]. Some of these bacteria synthesize CH_4_ using acetic acid, including the *Methanosaeta, Methanosarcina*, and *Methanothrix* genera. These are acetoclastic or acetotrophic methanogens. Additionally, other groups of methanogens synthesize CH_4_ by utilization of H_2_ and CO_2_ or methyl compounds, such as *Methanobacterium*, *Methanococcus*, *Methanospirillum*, or *Methanomassiliicoccus* [111].

These bacteria are potentially able to use all types of biomass suitable for producing biogas: sewage sludge from aerobic wastewater treatment, animal manure, harvest residues, organic waste from agriculture and food processing factories, dairy waste, organic fraction of municipal solid waste (OFMSW), fruit and vegetable waste, and energy crops, which are substrates commonly used for feeding anaerobic digesters [112].

The amount of biogas obtainable from a specific substrate depends on the operating conditions and its content of carbohydrates, proteins, and lipids. Lipids require a longer time than carbohydrates and proteins to be converted into biogas but have a more efficient conversion rate in terms of biogas produced per gram of substrate thanks to a high number of C and H atoms in their molecules [113]. Lipids are commonly present in food waste and in several wastewater types from factories, such as those that process meat, produce dairy, or refine fat [114]. Lipids can often be the cause of inconveniences, such as the inhibition of methanogenic microorganisms or their flotation and subsequent washout [115].

Organic waste from agriculture, food waste, and OFMSW is mainly composed of carbohydrates. Such wastes are easily degraded; if their feeding is not accurately controlled, volatile fatty acids (VFAs) produced by the acidification step of the anaerobic digestion tend to accumulate, causing a sharp drop in the pH value, which inhibits the activity of methanogenic Archaea [116] and leads to underperformance of the process.

Wastes rich in proteins are commonly produced by meat and fish processing factories, slaughterhouses, and farms (animal slurry and manure). These wastes are characterized by a low *C*/*N* ratio [117–119] that can hamper and even inhibit the activities of microorganisms [120]. Furthermore, proteins undergoing anaerobic digestion are converted into ammonia as an end product, which is rather toxic to microorganisms [121] and should be considered when looking for cost-effective ammonia removal techniques [122].

Wastes rich in cellulose (CWs) are produced by paper and cardboard as well as textile factories. CWs are also found, in large amounts, in unsorted municipal solid wastes (MSWs) and therefore are not useful for recycling. The *C*/*N* ratio in CWs is usually high, ranging from 173/1 up to values higher than 1000/1 [123], while the optimum *C*/*N* ratio ranges from 20/1 to 30/1 [124].

Microalgae can be an alternative substrate for renewable energy recovery. The co-digestion of microalgae with different types of wastes, such as pig/dairy manure [125], lipid waste (fat, oil, and grease) [126], waste activated sludge [127], and corn straw [128], has been extensively evaluated for biomethane production. Zhen et al. [129] examined the technical potential of methane production from microalgae through co-digesting with food waste. The results showed that supplementing food waste significantly improved microalgae digestion performance compared to the digestion of a single food waste, with the highest methane yield of 639.8 ± 1.3 ml g^−1^ VS_added_.

In fact, an estimation of the amount of methane that can be produced from a specific substrate is commonly obtained through a specific test called the biomethane potential test (BMP). The BMP can be used as an index of the anaerobic biodegradation potential, as it is the experimental value of the ultimate specific biomethane production for the indefinite degradation time [130]. However, in practice, BMP is estimated at a well-defined degradation time that can be a specific day, e.g., the 30th [131, 132] or 50th [133] of incubation or the day when biomethane production is approximately zero [134] or less than 5 mld^−1^ [131]. BMP can be expressed specifically as a volume of methane per amount of waste (dm^3^ CH_4_ kg^−1^ waste), volume of waste (dm^3^ CH_4_ dm^−3^ waste), per mass volatile solids added (dm^3^ CH_4_ kg^−1^ VS), or COD (chemical oxygen demand) added (dm^3^ CH_4_ kg^−1^ COD). The volume is usually expressed at standard conditions in terms of pressure (1 atm) and temperature (0 °C). Other units for expressing methane potential are also used [135].

For the same substrate, the BMP results can be variable because it is affected by the operating conditions in terms of temperature, mixing intensity, pH adjustment, substrate/inoculum (S/I) ratio, substrate particle size, liquid/volume ratio, nutrient content, inoculum, and if the substrate has been previously pretreated (e.g., mechanically, thermally, chemically) or mixed with one or more other substrates to perform a co-digestion process [136]. In Table 8, the methane yields from different substrates are reported (adapted from Raposo et al. [112]).

**Table 8:** Methane yields of solid organic substrates. Adapted from Raposo et al. [85]

Solid organic substrate	Methane yield [ml CH_4_ g^−1^ VS_added_]	References
Apple fresh wastes	317	[179]
Banana peeling	289	[179]
Cabbage leaves 2 mm size	309	[180]
Carrot peeling	388	[179]
Cauliflower leaves	341–352	[181]
Cellulose	356–375	[132]
Cocksfoot	325	[182]
Food wastes	245–510	[183]
Fruit and vegetable wastes	470	[184]
Glucose	335	[185]
Kitchen waste	432	[115]
Leather fleshing	490	[186]
Lettuce residues	294	[179]
Maize residues	317	[187]
Mandarin peels 2 mm size	486	[180]
OFMSW	353	[188]
Orange peeling	297	[179]
Paper and cardboard	109–128	[189]
Pineapple peel	400	[190]
Potato waste	320 referred to gVS_removed_	[191]
Rape oil seed	800–900	[133]
Rice straw	347–367	[192]
Starch	348	[133]
Sugar beet	340	[193]
Sunflower	428–454	[194]
Textiles	228	[195]
Tomato skins and seeds	218	[187]
Wheat straw	267	[196]
Algal biomass	640	[129]

### Biohydrogen production

Hydrogen is considered an ideal source of energy because it represents a clean combustible and is also easily convertible to electricity [137]. Biological hydrogen production is related to biogas production for two main reasons: a similar production process, and the same substrates are suitable. These two gaseous products are derived from the same biological process that switches on hydrogen production when hydrogen-using microorganisms are inhibited, such as homoacetogens and methanogens; inhibition is commonly achieved through heat treatment of the inoculum to remove all microorganisms, except for spore-forming fermenting bacteria (i.e., species belonging to the families *Clostridiaceae*, *Streptococcaceae*, *Sporolactobacillaceae*, *Lachnospiraceae,* and *Thermoanaerobacteriacea*) [138]. The most common bacteria used in dark fermentation to produce hydrogen are *Clostridium* [139] and *Thermoanaerobacterium* [140, 141]. Moreover, several studies have reported successful hydrogen production by mixed cultures in batch or bioreactors [142, 143]. The advantages of using mixed cultures for biohydrogen production are several and are as follows: no need for sterilization, a high adaptive capacity owing to the microbial diversity, the capacity to use a mixture of substrates, and the possibility of obtaining a stable and continuous process [141].

Furthermore, the same organic substrates, such as solid waste, can be used to produce biogas and biohydrogen, thus converting residues into a source of bio-energies [138]. Many processes for hydrogen production have been extensively investigated; among them, hydrogen production by photosynthetic bacteria, algae, and fermentative bacteria is the most interesting because it is environmentally sustainable.

In autotrophic conversions, biohydrogen can be produced by photosynthetic microorganisms, i.e., microalgae and photosynthetic bacteria that convert solar energy to hydrogen [144]. Photosynthetic bacteria (e.g., purple non-sulfur bacteria) utilize the end products of dark fermentation, converting them into H_2_ via photofermentation with simultaneous VFA reduction [145–150]. The major limitation of photofermentation systems is its poor H_2_ production rate due primarily to the slow growth of photosynthetic bacteria and the low light conversion efficiency of photobioreactors [149]. A photobioreactor (PBR) was developed by Chen et al. [149] to enhance phototrophic H_2_ production by *Rhodopseudomonas palustris* WP3-5 using acetate as the sole carbon source. The photobioreactor was illuminated by combinative light sources, reaching a maximum H_2_ yield of 62.3%.

Under heterotrophic conditions, two types of fermentation occur: photofermentation carried out by photosynthetic bacteria and dark fermentation [151] carried out by anaerobic bacteria that convert carbohydrates into biohydrogen [144]. Different rumen bacteria, such as *Clostridia*, methylotrophs, methanogenic archae, or facultative anaerobic bacteria (*E. coli*, *Enterobacter* spp.*, Citrobacter* spp.), and aerobic bacteria (*Alcaligenes* spp., *Bacillus* spp.) have been studied to perform dark fermentation. In particular, *Clostridium butyricum* and *Clostridium articum* produce butyric acid and propionate as major products, respectively, and both products are of interest for hydrogen production [152]. Indeed, photofermentation takes place under anaerobic conditions involving purple non-sulfur photosynthetic bacteria using light as an energy source for synthesizing hydrogen [153]. The ability of purple non-sulfur bacteria to convert organic acids to biohydrogen is coupled with their ability to synthesize PHB under anaerobic conditions.

In fact, Luongo et al. [154] investigated hydrogen and poly-b-hydroxybutyrate (PHB) production during photofermentative treatment of the effluent from a dark fermentation reactor fed with the organic fraction of municipal solid waste. They compared the hydrogen and PHB production of an adapted culture of *Rhodobacter sphaeroides* AV1b and a mixed consortium of purple non-sulfur bacteria. The mixed cultures resulted in 1.5-fold more H_2_ produced than the pure culture (559 and 364 N ml H_2_ l^−1^, respectively). On the contrary, *R. sphaeroides* cultures showed higher PHB productivity (155 mg PHB g COD^−1^) than the mixed cultures (55 mg PHB g COD^−1^).

As for methane production through anaerobic digestion, biohydrogen can be produced by different bacterial strains using several organic substrates. For example, Cappelletti et al. [155] focused their study on H_2_ production from molasses and cheese whey with the aim of valorizing food industry wastes by their recycling; mesophilic, thermophilic, and hyperthermophilic bacteria were tested to produce H_2_. Among them, *Thermotoga* strains showed the most promising results; in particular, *T. neapolitana* was the best performing strain (Table 9). This result was confirmed by studies conducted on *T. neapolitana* using other organic substrates, such as rice straw [156], beet pulp pellet, corn starch, and rice flour [157]. Such substrates are particularly suitable for producing H_2_ thanks to their easy biodegradability and are also convenient because they are present in different carbohydrate-rich wastewaters and agricultural residues [158]. Other substrates commonly used for biohydrogen production are protein- and fat-rich wastes. A *C. butyricum* strain was studied by Chen et al. [159] for its ability to produce H_2_ from a sucrose-based medium. In particular, *C. butyricum* CGS5 can efficiently produce hydrogen (2.78 mol H_2_ mol^−1^ sucrose) on an iron-containing medium [159]. The same microbial strain (*C. butyricum* CGS5) was isolated from soil with nine cellulolytic bacterial strains belonging to *Cellulomonas* sp. and *Cellulosimicrobium cellulans* by Lo et al. [160]. Among these strains, only *C. butyricum* CGS5 exhibited efficient H_2_ production from rice husk hydrolysates, with a H_2_ yield of 17.24 mmol H_2_ g cellulose^−1^.

**Table 9:** Hydrogen yields of different substrates. Adapted from Li and Fang [141], Davila-Vazquez et al. [158]

Substrate	Strain	Hydrogen yield	References
Sucrose	*Clostridium butyricum* CGS5	2.78 [mol H_2_ mol^−1^ substrate]	[159]
Glucose	*Escherichia coli* strains	2 [mol H_2_ mol^−1^ substrate]	[162]
Glucose	*Thermotoga neapolitana*	1.6 [mol H_2_ mol^−1^ substrate]	[155]
Molasses	*Thermotoga neapolitana*	2.6 [mol H_2_ mol^−1^ substrate]	[155]
Rice straw	*Thermotoga neapolitana*	2.7 [mol H_2_ mol^−1^ substrate]	[156]
Cheese whey	*Thermotoga neapolitana*	2.4 [mol H_2_ mol^−1^ substrate]	[155]
Cheese whey	*Clostridium saccharoperbutylacetonicum* ATCC 27021	2.7 [mol H_2_ mol^−1^ substrate]	[161]
Starch	Mesophilic bacterium HN001	2 [mol H_2_ mol^−1^ substrate]	[163]
Starch	Mixed culture from compost	133 [ml H_2_ g^−1^ hexose]	[166]
Cellulose	Mixed culture from sludge	92 [ml H_2_ g^−1^ hexose]	[164]
Mixed waste	Mixed culture from anaerobic digestion sludge	201 [ml H_2_ g^−1^ hexose]	[168]
Food waste	Mixed culture from anaerobic digestion sludge	210 [ml H_2_ g^−1^ hexose]	[167]
Acetate	*Rhodopseudomonas palustris* WP3-5 in Photobioreactor	62.3 [mol H_2_ mol^−1^ substrate]	[120]
Rice husk	*Clostridium butyricum* CGS5	17.24 [mmol H2 g^−1^ cellulose]	[160]

Ferchichi et al. [161] investigated hydrogen production from cheese whey by *Clostridium saccharoperbutylacetonicum*, studying the influence of the initial pH; they found that slightly acidic initial conditions favored a higher H_2_ yield than alkaline conditions. The highest hydrogen yield (2.7 mol H_2_ mol^−1^ substrate) was actually obtained at pH 6. Bisaillon et al. [162] examined hydrogen production by different strains of *Escherichia coli* under different feeding regimes to detect the main limiting factors: strains that showed the highest hydrogen yield (2 mol H_2_ mol^−1^ substrate) when cultured at limiting concentrations of either ammonia or glucose (1 mM NH_4_Cl; 0.04% of glucose). Mesophilic bacterium HN001 was tested by Yasuda and Tanisho [163] as a H_2_ producer from starch. In the same work, the authors focused their studies on the influence of temperature, pH, and substrate concentration; the optimal temperature was found to be approximately 37 °C, with a hydrogen yield of 2 mol H_2_ mol^−1^ substrate. Liu et al. [164] investigated H_2_ production by mixed cultures in batch experiments using cellulose as a substrate; at the optimal pH of 6.5, the maximum hydrogen yield was 92 ml H_2_ g^−1^ hexose, and an analysis of 16S rDNA sequences showed that the cellulose-degrading mixed culture was composed of microbes closely affiliated with genus *Thermoanaerobacterium*. Carbohydrate-rich holocellulose of lignocellulosic organic matter can be made available to the H_2_ conversion by pretreatment. Examples of lignocellulosic biomass pretreatment methods for hydrogen fermentation were reported by Kumar et al. [165]. They also reported the maximum hydrogen yield associated with pretreatment methods, ranging from 44.9 to 141.29 ml H_2_ g^−1^.

The influence of pH was also evaluated by Khanal et al. [166], who used a mixed microbial culture and starch as a substrate. At the optimal pH of 4.5, the maximum hydrogen yield was 133 ml H_2_ g^−1^ hexose. At the same pH value, Fang et al. [167] reached a maximum hydrogen yield of 210 ml H_2_ g^−1^ hexose using food waste as a substrate. Instead, Valdez-Vazquez et al. [168] studied the influence of temperature using a mixed culture as the inoculum and mixed waste as a substrate. At 37 °C, the maximum hydrogen yield was 210 ml H_2_ g^−1^ hexose.

All biotechnological hydrogen production processes have particular limits, since a considerable part of the used substrate is converted into various soluble metabolic products rather than H_2_. Thus, the major side product of dark fermentation is a multi-compound mixture of VFAs and other constituents, such as alcohols [169]. Therefore, the volatile fatty acid-rich fermentation effluent is a perfect substrate for biologically synthesizing polyesters, e.g., polyhydroxyalkanoate [170, 171], which could have an industrial market [172].

## Integrated systems for bioenergy production from industrial and agricultural wastes

### Simultaneous production of PHAs and bioenergy from organic wastes

Degradation of biowaste to methane (CH_4_) and carbon dioxide is a multiple step process with the possibility of producing H_2_ and bioplastics (from volatile fatty acids) as intermediates [17]. Based on this process, anaerobic digestion can be performed with a two-stage system, where biomass is degraded in the first stage and hydrolysis–acidification occurs. The organic acids produced are processed under aerobic conditions to produce biopolymers and, as an alternative, under anaerobic conditions to produce biogas.

**Fig. 3:**
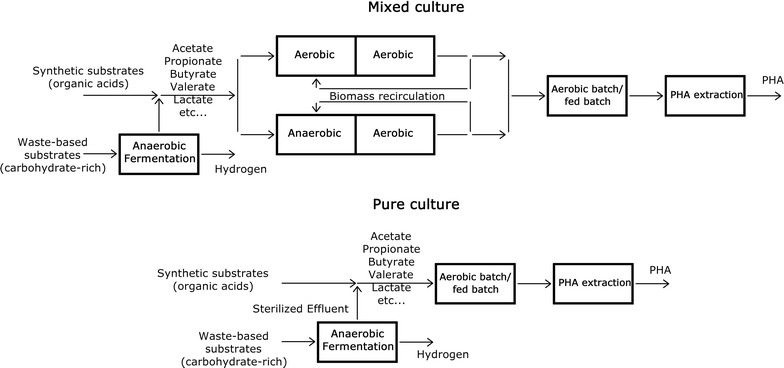
Cycle of polyhydroxyalkanoates (PHAs) production system (Adapted from Serafim et al. [173])

A PHA production system, in its most comprehensive configuration, is composed of four main stages (Fig. 3), as follows:

1. Feedstock production,
2. Biomass selection,
3. PHA production, and
4. PHA extraction.

Simplified configurations can be obtained using synthetic substrates (stage 1 is removed from the cycle), using pure culture (stage 2 is removed from the cycle), or using both synthetic substrates and pure culture (stages 1 and 2 are removed from the cycle).

The aims of each stage are listed below:

1. To produce organic acids from complex organic solids (e.g., wastes rich in carbohydrates),
2. To select the microbial strains from the mixed culture that show the highest capacity for PHA accumulation under specific dynamic feeding conditions [173],
3. To produce PHAs using the selected culture, and
4. To recover PHAs from microorganisms.

A dark fermentation process can be successfully used to perform the first stage. This process evolves according to the same sequence of biochemical reactions in the anaerobic digestion process, with the exception of the last stage that is repressed using different strategies (e.g., setting a short hydraulic retention time-HRT, keeping the pH low at 5.5, adding chemical compounds toxic to methanogens, and performing thermal shocks).

**Fig. 4:**
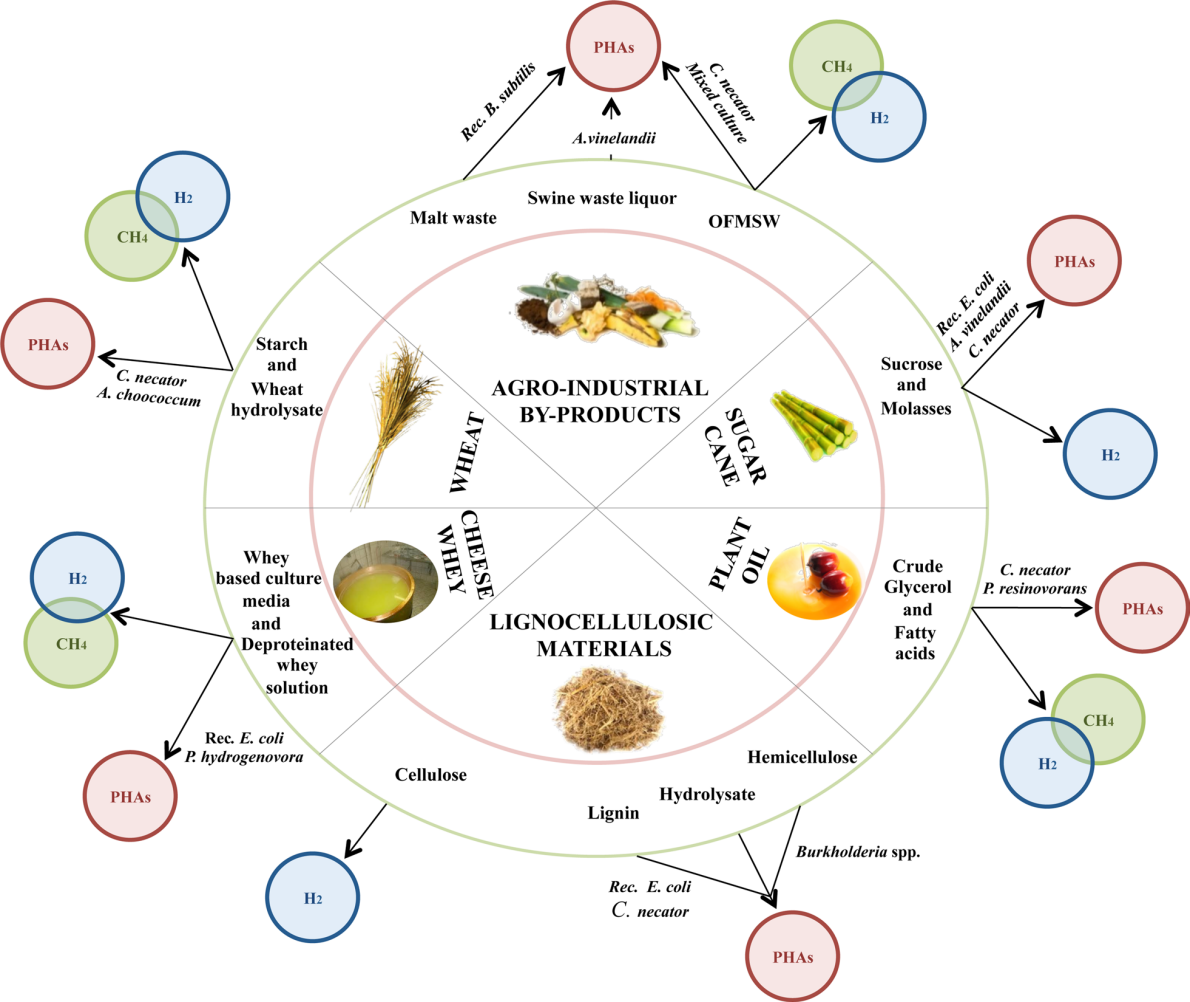
Sustainable PHAs and bioenergy production from organic wastes and by-products converted by different bacterial species: an overview of the principal process considered in this review

The dark fermentation process can be optimized to produce VFAs and consequently H_2_ that is a by-product of the biological process and VFAs, varying (i) the operational conditions (i.e., pH, temperature, HRT, solid retention time—SRT, organic loading rate—OLR); (ii) the configuration of the dark fermentation reactor and feeding system; and (ii) the type of organic waste used to feed the reactor (Fig. 4). The effects of these parameters on VFA production are listed in Table 10 [174].

**Table 10:** Waste, reactor configuration, and operation for the production of VFAs Adapted from Lee et al. [174]

Type of waste	Organic content (mg COD l^−1^)	Reactor type and operating conditions	VFA production	References
Waste activated sludge	18.657	Batch, 55 °C, pH = 8, HRT = 9 days	368 mg COD gVSS^−1^	[197]
	14.878	Batch, 21 °C, HRT = 6 days	339 mg COD l^−1^	[198]
Primary sludge	22.838	Batch, 21 °C, HRT = 6 days	85 mg COD gVSS^−1^	[199]
	20.631	Batch, 21 °C, pH = 10, room temp, HRT = 5 days	60 mg COD gVSS^−1^ day^−1^	[200]
Food waste	91.900	Batch, 37 °C, pH = 5.5	8950 mg COD l^−1^	[201]
	146.1	Batch, 35 °C, HRT = 5 days	5610 mg COD l^−1^	[202]
Kitchen waste	166.18	Batch, 35 °C, pH = 7, HRT = 4 days	36 mg l^−1^	[203]
OFMSW	347.0	Batch, 14–22 °C, pH = 4–5, HRT = 4–4.5 days	40 mg g^−1^ VS	[204]
	196.7	Plug flow, 37 °C, pH = 5.7–6.1, HRT = SRT = 6 days, OLR = 38.5 gVS l^−1^ day^−1^	23.110 mg l^−1^	[15]
Palm oil mill	88.0	Semi-continuous, 30 °C, pH = 6.5, HRT = 4 days	15.300 mg l^−1^	[205]
Olive oil mill	37.0	Packed bed biofilm, 25 °C, pH = 5.2–5.5, HRT = 14 days, OLR = 26 g COD l^−1^ day^−1^	10.700 mg COD l^−1^	[206]
Cheese whey	4590	CSTR, 37 °C, pH = 6, HRT = 2.1 days	0.84 gVFA-COD g^−1^ sCOD^−1^	[207]

Various microbes, such as *A. eutrophus, B.s megaterium, P. oleovorans, A. beijerincki, Rhizobium*, and *Nocardia*, utilize acetic acid, formic acid, and propionic acid as a substrate for PHA production [70]. *A. eutrophus* and *A. beijerinckii* were studied by Kalia et al. [70] and were shown to be capable of accumulating PHAs up to 70% of CDW, under nitrogen and phosphorus-limiting conditions, whereas *Pseudomonas* spp. and *Rhizobium* spp. accumulated PHAs at approximately 60% of CDW.

Many other bacterial strains have also been reported to produce PHAs under adverse conditions with different PHA yields. Among them, many purple non-sulfur bacteria, such as *R. sphaeroides*, *Rhodospirillum rubrum*, *Rhodopseudomonas palustris*, *Rhodopseudomonas palustri*s, and *Bacillus* spp., have been reported to produce H_2_ and PHA under nutrient-limiting conditions [175].

Patel et al. [17] investigated the metabolic activities of *Bacillus* strains to transform glucose into H_2_ and PHB in two stages. Operating in batch mode, *Bacillus thuringiensis* EGU45 and *B. cereus* EGU44 reached 1.67–1.92 mol H_2_ mol^−1^ glucose, respectively, during the first 3 days. In the next 2 days, *Bacillus thuringiensis* EGU45 was supplemented with residual medium containing glucose, volatile fatty acids, and residual nutrients (nutrient stress condition) and produced a PHB yield of 11.3% of CDW.

*Rhodopseudomonas palustris* WP3-5 was studied by Wu et al. [176] to evaluate possible competition between PHB synthesis and H_2_ production, testing cultures on six different substrates, such as acetate, propionate, malate, lactate, glucose, and lactose. The results highlighted that strain WP3-5 could utilize acetate, propionate, malate, and lactate to produce H_2_, whereas it was also able to synthesize PHB only on acetate and propionate. PHB synthesis decreased H_2_; however, under pH-stress conditions, such a decrease was not observed.

*Rhodopseudomonas palustris* was also studied by Vincenzini et al. [177] to investigate the potential of purple non-sulfur bacteria in the photoproduction of both hydrogen and PHB-containing biomass under limiting amounts of nitrogen. The data demonstrated that under nitrogen-limiting growth conditions, *R. palustris* synthesized 40 mg l^−1^ day^−1^ of PHB and produced 200 ml l^−1^ day of H_2_ when the experiments were supplemented with 60 mg l^−1^ day^−1^ of nitrogen.

Yu [26] performed a two-step integrated system consisting of microbial acidogenesis and acid polymerization from starchy wastewater. In his work, the starchy organic waste was first digested in a thermophilic upflow anaerobic sludge blanket reactor to form acetic (60–80%), propionic (10–30%), and butyric (5–40%) acids. The acids in the effluent solution after microfiltration were polymerized into PHAs by *A. eutrophus* in a second reactor. PHA production from the acid effluent was compared with the production from pure acids in 48 h, and the results were very similar. In batch mode, 1.2 g l^−1^ of PHAs was accumulated from acid effluent. Instead, 1.0 and 1.3 g l^−1^ of PHAs were obtained from a mixture of butyric acid and propionic acid in batch and fed-batch mode, respectively.

Albuquerquea et al. [178] designed another integrated system to valorize the use of wastewater for PHA production. They employed a 2-stage continuous stirred tank reactor (CSTR) system to effectively select PHA-storing organisms using fermented molasses as feedstock. The acidogenic fermentation (step 1) was carried out in a CSTR operated under anaerobic conditions. The reactor effluent was clarified by microfiltration and used as a feedstock for culture selection (step 2) and PHA batch accumulation (step 3). The culture reached a maximum PHA content of 61%.

The best integrated system developed was based on two-step processes consisting of acidogenic fermentation (operating under anaerobic condition) aimed to produce acid effluent that, after microfiltration, is used in the subsequent aerobic microbial process aimed at PHA polymerization. However, the first step (acidogenic fermentation) is also useful for hydrogen production and could be designed as a dark fermentation process.

## Conclusions

Biological processes can be successfully used in innovative and eco-sustainable technology to convert organic waste into bioenergy and biochemicals, separately or simultaneously. Bioprocesses can provide bioenergy or valuable chemicals and, at the same time, perform pollution control, according to technical feasibility, simplicity, economics, and societal needs. Bio-based plastics can completely replace the conventional ones derived from fossil fuels if the production costs can be reduced, and the use of high-performing bacteria fed with organic wastes and by-products as substrates significantly contribute to achieving this objective.

In this context, different organic substrates and by-products can be used to produce bioenergy (hydrogen and methane) and biopolymers (PHAs). Otherwise, the review highlights the possibility of integrating the two production processes to design a unique system for both energy and biopolymer production. The integrated system is a flexible process that aims (i) to produce organic acids from complex organic solid wastes rich in carbohydrates; (ii) to use selected microbial strains or mixed cultures that show the highest capacity for PHA accumulation under specific dynamic feeding conditions; and (iii) to produce bioenergy or accumulate PHAs by microorganisms from acidogenic effluents.

This integrated system represents new perspectives on the use of organic waste and by-products, valorizing organic substrates for the production of both bioenergy and PHAs.

## Abbreviations

OFMSW: organic fraction of municipal solid waste
PHAs: polyhydroxyalkanoates
EPS: exopolysaccharides
GHG: green house gas
SCL: short-chain length
MCL: medium-chain length
P(3HB): poly(3-hydroxybutyrate)
PHB: polyhydroxybutyrate
3HV: 3-hydroxyvalerate
4HB: 4-hydroxybutyrate
P(3HB-3HV): poly(3-hydroxybutyrate-co-3-hydroxyvalerate)
LCA: life cycle assessment
CDW: cell dry weight
*C*/*N*: carbon-to-nitrogen ratio
PHBV: poly(hydroxybutyrate-co-hydroxyvalerate)
UASB: upflow anaerobic sludge blanket
AO: alginate oligosaccharides
VFAs: volatile fatty acids
CWs: cellulose wastes
BMP: biomethane potential test
COD: chemical oxygen demand
S/I: substrate/inoculums ratio
HRT: hydraulic retention time
SRT: solid retention time
OLR: organic loading rate
